# A randomised controlled trial of clinical and cost-effectiveness of the PASS Plus intervention for young children with autism spectrum disorder in New Delhi, India: study protocol for the COMPASS trial

**DOI:** 10.1186/s13063-023-07621-5

**Published:** 2023-10-12

**Authors:** Reetabrata Roy, Kathy Leadbitter, Gemma Shields, Carol Taylor, Catherine Aldred, Monica Juneja, Sheffali Gulati, Vivek Vajaratkar, Linda Davies, Richard Emsley, Vikram Patel, Gauri Divan, Jonathan Green, the COMPASS team

**Affiliations:** 1 Sangath, Porvorim, India; 2 University of Manchester, Manchester, UK; 3 Maulana Azad Medical College and Associated Lok Nayak Hospital, Delhi, India; 4 All India Institute of Medical Sciences, Delhi, India; 5 Goa Medical College, Panaji, India; 6 Department of Biostatistics and Health Informatics, Institute of Psychiatry, Psychology and Neuroscience, King’s College London, London, UK; 7 Harvard Medical School, Boston, USA; 8 Royal Manchester Children’s Hospital, Manchester, UK

**Keywords:** Autism spectrum disorder, Randomised controlled trial, Parent-mediated intervention, Non-specialist workers, Cost-effectiveness

## Abstract

**Background:**

Autism Spectrum Disorder (ASD) is a neurodevelopmental disability affecting at least 5 million children in South Asia. Majority of these children are without access to evidence-based care. The UK Pre-school Autism Communication Therapy (PACT) is the only intervention to have shown sustained impact on autism symptoms. It was systematically adapted for non-specialist community delivery in South Asia, as the ‘Parent-mediated Autism Social Communication Intervention for non-Specialists (PASS)’ and extended ‘PASS Plus’ interventions. RCTs of both showed feasibility, acceptability and positive effect on parent and child dyadic outcomes.

**Methods:**

The Communication-centred Parent-mediated treatment for Autism Spectrum Disorder in South Asia (COMPASS) trial is now a scale-up two-centre, two-arm single (rater) blinded random allocation parallel group study of the PASS Plus intervention in addition to treatment as usual (TAU) compared to TAU alone, plus health economic evaluation embedded in the India health system. Two hundred forty children (approximately 120 intervention/120 TAU) with ASD aged 2–9 years will be recruited from two tertiary care government hospitals in New Delhi, India. Accredited Social Health Activists will be one of the intervention delivery agents. Families will undertake up to 12 communication sessions over 8 months and will be offered the Plus modules which address coexisting problems. The trial’s primary endpoint is at 9 months from randomisation, with follow-up at 15 months. The primary outcome is autism symptom severity; secondary outcomes include parent–child communication, child adaptation, quality of life and parental wellbeing. Primary analysis will follow intention-to-treat principles using linear mixed model regressions with group allocation and repeated measures as random effects. The cost-effectiveness analysis will use a societal perspective over the 15-month period of intervention and follow-up.

**Discussion:**

If clinically and cost-effective, this programme will fill an important gap of scalable interventions delivered by non-specialist health workers within the current care pathways for autistic children and their families in low-resource contexts. The programme has been implemented through the COVID-19 pandemic when restrictions were in place; intervention delivery and evaluation processes have been adapted to address these conditions.

**Trial registration:**

ISRCTN; ISRCTN21454676; Registered 22 June 2018.

## Administrative information

Note: the numbers in curly brackets in this protocol refer to SPIRIT checklist item numbers. The order of the items has been modified to group similar items (see http://www.equator-network.org/reporting-guidelines/spirit-2013-statement-defining-standard-protocol-items-for-clinical-trials/).

**Table:** 

**Title {1}**	A randomised controlled trial of clinical and cost-effectiveness of the PASS Plus intervention for young children with autism spectrum disorder in New Delhi, India: Study protocol for the COMPASS trial.
**Trial registration {2a and 2b}.**	ISRCTN ID: 21,454,676 https://www.isrctn.com/ISRCTN21454676?q=21454676 Registration date: 22.06.2018
**Protocol version {3}**	03.08.2022; Version 4
**Funding {4}**	Joint Global Health Trials Programme; Medical Research Council (MRC), the Department of Health and Social Care (DHSC), the Foreign, Commonwealth & Development Office (FCDO), and Wellcome Trust (WT). Grant reference: MR/R006164/1.
**Author details {5a}**	1. Reetabrata Roy r.roy@sangath.in, Sangath, India (Corresponding author; ORCID ID: 0000–0002-1430-369X) 2. Kathy Leadbitter, kathy.leadbitter@manchester.ac.uk, University of Manchester, UK 3. Gemma Shields, gemma.shields@manchester.ac.uk, University of Manchester, UK 4. Carol Taylor, carol.taylor-2@manchester.ac.uk, University of Manchester, UK 5. Catherine Aldred, catherine@pacttraining.co.uk, University of Manchester, UK 6. Monica Juneja, drmonicajuneja@gmail.com, Maulana Azad Medical College and associated Lok Nayak Hospital, India 7. Sheffali Gulati, sheffaligulati@gmail.com, All India Institute of Medical Sciences, India 8. Vivek Vajaratkar, vivek.vajaratkar@sangath.in, Goa Medical College, India 9. Linda Davies, linda.m.davies@manchester.ac.uk, University of Manchester, UK 10. Richard Emsley, richard.emsley@kcl.ac.uk, Kings College London, Department of Biostatistics and Health Informatics, Institute of Psychiatry, Psychology and Neuroscience, UK 11. Vikram Patel, Vikram_Patel@hms.harvard.edu, Harvard Medical School, USA 12. Gauri Divan, gauri.divan@sangath.in, Sangath, India 13. Jonathan Green, jonathan.green@manchester.ac.uk, University of Manchester & Royal Manchester Children’s Hospital, Manchester, UK
**Name and contact information for the trial sponsor {5b}**	Mohammed Zubair Research Governance, Ethics and Integrity Manager The University of Manchester 0161 275 2725 mohammed.zubair@manchester.ac.uk
**Role of sponsor {5c}**	The study sponsor and funders played no role in: the study design; the collection, management, analysis and interpretation of data; the writing of the report; and the decision to submit for publication.

## Introduction

### Background and rationale {6a}

Over 80% of children with Autism Spectrum Disorder (ASD) live in Low and Middle Income Countries (LMICs), and at least 5 million children in South Asia are autistic, the great majority without access to evidence-based care. This lack of access to evidence-based care along with the stigma and discrimination faced by families of children with ASD [1] impacts parental well-being and their quality of life [2]. Recent evidence from high-income countries supports the effectiveness of targeted parent-mediated interventions for the early care of autistic children and studies from Southeast Asia reflects that parents receiving interventional support report a better quality of life [3, 4]. Interventions that are delivered through parents have the additional advantages of improving parental knowledge and wellbeing, potentially promoting the social empowerment of mothers, generalising into improvements in the family environment for the child and thus potentially conferring long-term impacts on the social context, the child’s environment and functional outcomes.

### UK evidence

The Pre-school Autism Communication Therapy (PACT) is the only intervention in the field so far to have shown sustained impact on autism symptoms [5]. The therapy derives from theory-based research into autistic development and is targeted at getting parents to recognise their child’s social communication differences and create an environment which gives the child space and time to communicate at their own pace. This intervention uses video feedback techniques to work with parents to enhance their understanding and responsiveness to the atypical communications of their young autistic child.

The MRC-funded UK PACT trial [5] (*N* = 152) and its 6-year follow-up at mean age 10.5 years [6] showed treatment effect to reduce autism symptom severity, both at the primary endpoint (ES 0.64, 95% CI 0.07, 1.20; a 15% relative reduction) and at follow-up (ES 0.70, 95% CI − 0.05, 1.47), with a statistically significant averaged effect over the whole period (ES 0.55, 95% CI 0.14, 0.91). The endpoint change was strongly mediated through the targeted optimisation of parent–child dyadic social communication [7], supporting the intervention theory: autistic children who received this treatment benefited from the enriched communication environment that their parents were able to create; this in turn had a long-term positive impact on the social interactions the children initiated and on the children’s autism symptoms. PACT is an NIHR ‘Signal’ study; the intervention is evidenced in UK NICE [8] and has been selected for implementation in the UK Department of Health Improving Access to Psychological Therapy (IAPT) program.

### Adaptation to South Asia

The original UK PACT therapy was adapted for use in South Asia in a systematic manner, including manual translation and cultural adaptation to enhance parental acceptability, developing a supervision and training cascade led by specialists in UK, India and Pakistan. Key additional features were to allow the adapted intervention to be delivered by community based non-specialist health workers and the delivery of the intervention at home. The resulting intervention was originally published as ‘Parent-mediated intervention for Autism Spectrum Disorders in South Asia’ but has now been renamed for use including beyond South Asia, as the ‘Parent-mediated Autism Social communication Intervention for non-Specialists’ (PASS) involved 12 sessions delivered over 8 months to children aged 2 to 9 years. A pilot RCT in India and Pakistan (the PASS study; 2012–2014; Autism Speaks) of the PASS intervention vs usual care (*n* = 65) [9] showed (i) high acceptability, with 24/32 (80%) of parent–child dyad completing all intervention sessions including 100% (10/10) of families from low socio-economic backgrounds; (ii) high therapist fidelity against original PACT criteria; and (iii) replication of dyadic PACT results with effect on parental (ES 0.25, 95% CI 0.14, 0.36) and child (ES 0.14, 95% CI 0.04, 0.24) dyadic social communication. The PASS intervention was showcased at the World Bank-WHO summit on mental health (Washington, April 2016) and at the World Innovation Summit for Health (Qatar, November 2016).

### Extension to co-occurring conditions

Subsequently, the team in India developed and piloted an enhancement of PASS which included a clinical decision algorithm containing strategies to address common co-occurring conditions in autistic children. These included sensory-seeking and disruptive behaviours (identified in the PASS trial as potential barriers to implementation and acceptability in low-resource settings), creating a comprehensive intervention for children with ASD in the 2–9-year age group (PASS Plus; PASS +). The pilot RCT of PASS + (The PASS + study; 2014–2016; Grand Challenges Canada) [10] demonstrated feasibility by meeting its recruitment target of 40 children in a rural setting; the intervention was delivered successfully with fidelity to 19 families randomised to intervention. Eighty-nine percent of intervention families partially or entirely completed the 12-session intervention. Intention-to-treat analysis showed a reduction in mean scores of autism symptom severity, though the confidence interval contained zero (adjusted mean difference [AMD] 2.42; 95% CI − 7.75, 2.92; ES 0.22); large treatment effects on proximal outcomes of proportion of parent synchronous responses (AMD 0.35; 95% CI 0.18, 0.52; effect size ES 3.97) and proportion of child communication initiations with parent (AMD 0.17; 95% CI 0.03, 0.32; ES 1.02). Confidence intervals for effects on mutual shared attention (AMD 0.10; 95% CI − 0.07, 0.27; ES 0.5) and co-morbid symptoms (AMD − 9.0; 95% CI − 24.26, 6.26; ES 0.32) contained zero.

Both these projects have enabled the India team to capacity build in training and supervision of non-specialist health workers. Evaluation methods have also been adapted and tested in both of these pilot studies, including the Brief Observation of Social Communication Change (BOSCC), the primary outcome measure to be used in COMPASS. The project team in India will preserve competence in delivery, analysis and dissemination seamlessly into COMPASS, minimising the risk of loss of capacity.

### Current trial

The proposed Communication-centred Parent-mediated treatment for Autism Spectrum Disorder in South Asia (COMPASS) trial will build on this pilot work already carried out in India and will constitute the largest definitive trial of the intervention, involving 240 participants recruited through two government-run tertiary care super-speciality hospitals linked to medical schools in the capital city of New Delhi, which cater across all populations with a significant representation of the urban poor. The intervention will take work conducted in the two previous trials to the next step by delivering through existing health system frontline workers to ensure that the intervention is scalable through existing human resources. This trial will be conducted in the capital city of New Delhi, which has 11 districts. As well as clinical effectiveness, the cost-effectiveness of intervention will be investigated. Economic evaluations support decision-making where there is growing demand placed on the healthcare system but with limited budgets, by synthesising costs and health benefits to allow for an assessment of cost-effectiveness (value for money).

Two groups of frontline workers—accredited social health activists (ASHA) and auxiliary nurse midwifes (ANM)—are deployed in the New Delhi National Urban Health Mission, through the primary urban health centres (PUHC). The PUHC covers a population of 50,000 residents, which is serviced by 3–4 ANMs and up to 25 ASHAs. The ASHA is a resident of the community and is a married woman between 25 and 45 years old. She is a bridge between the local community and the health services overseeing a population of 2000. Her role includes supporting the organisation of urban health and nutrition days, motivating families for institutional deliveries and accompanying mothers to institutions during childbirth, conducting home visits during pregnancy and for the new-born over the first month of life, mobilising families to attend immunisation days, supporting them to introduce appropriate complementary feeds and encouraging the adoption of family planning methods and help for medical illnesses along with being the frontline worker for various vertical programs (e.g. supporting environmental hygiene around infectious diseases). She is supervised by the ANM who is the key frontline health worker at the PUHC. The ANM supports maternity care as her primary role. The COMPASS team will work with the State Health Mission to identify ASHA workers spread across a geographical area suitable to our recruitment centres, to deliver the COMPASS intervention. The formative phase of the trial, particularly the mapping of children referred from the recruitment site, allowed us to understand the distribution of high-density clusters of families. Simultaneously, we were able to determine that the time available to the ASHA worker for COMPASS work would be limited. To address the possible risk to the completion of the trial intervention cases in these areas, the trial steering committee in January 2019 supported the plan of using a mixed worker model. Since mapping of cases from the recruitment site has revealed that there are 9 districts (of 11 districts in New Delhi) which have a clustering of families, we will focus on identifying and recruiting ASHA workers from each of these districts. Simultaneously, we will identify ASHA like workers who will be project appointed workers but will share the same characteristics as the ASHA workers (e.g. age, education level and local residence). These collectively will be known as COMPASS counsellors and will be trained and supervised in the same manner. This mixed worker model will have COMPASS counsellors trained to deliver to trial families.

The trial will evaluate the clinical and cost-effectiveness of the intervention on symptoms of ASD and parent–child interaction as well as more general impacts on child functioning, parental well-being and social empowerment. COMPASS will be the largest trial of its kind for ASD in any LMIC setting, and the evidence generated will have an impact not only on health policy and practice in India but also other low-resource settings in the region.

## Objectives {7}

1. To evaluate the effectiveness at scale of a parent-mediated intervention for autism spectrum disorders in South Asia, delivered by non-specialists in community health settings
2. To investigate the cost-effectiveness of the intervention compared to TAU
3. To generate tools and evidence for policy makers to guide the scale up the intervention

## Trial design {8}

The trial design is as follows: two-centre, two-arm single (rater) blinded random allocation parallel group superiority trial [11] of experimental treatment plus treatment as usual (TAU) against TAU alone. The primary endpoint is at 9 months from randomisation, with follow-up at 15 months. The primary outcome is autism symptom severity, as measured by the blind-rated Brief Observation of Social Communication Change (BOSCC). Secondary outcomes include parent–child communication, child adaptation and quality of life and parental wellbeing.

## Methods: participants, interventions and outcomes

### Study setting {9}

The trial will take place in a population representative of the urban disadvantaged, a fast-growing demographic who represent an under-researched and under-served population in India and in global health. Whilst an urban elite can access multi-disciplinary private care from community-based NGOs and private providers, the urban disadvantaged must rely entirely on busy government tertiary hospital paediatric services. The latter is our TAU and represents the ‘best available’ care in the public sector; this is delivered from autism clinics in specialist child development centres, which provide a programme of eclectic behavioural therapy approaches. However, take up is patchy and often compromised by the need to travel to these specialist centres; adherence is under 50%. In short, this TAU is highly expensive and not scalable due to its complete reliance on tertiary hospital-based specialists. Systematic data on precise treatment uptake is lacking (it will be provided by the health economic aspect of this trial), but the aim of the experimental model is to remedy the deficiencies of care for this important demographic by providing a home-based individualised evidenced and quality service in an efficient step-down fashion, using novel digital support (described below).

#### Implementation partner

Sangath (www.sangath.com) who will be the primary implementing partner in India is a non-profit research organisation with its headquarters in Goa and offices in New Delhi. It was founded in 1996 by the co-PI (Vikram Patel) and others. Sangath was awarded the prestigious MacArthur Foundation International Prize for Creative and Effective Institutions in 2008 and the WHO Public Health Champions Award for India in 2016. It has completed a number of randomised controlled trials for mental disorders in India, including the only two autism trials in the country and the largest trial in psychiatry in LMIC [12]. It was the State Nodal Agency for the National Trust for the Welfare of Persons with Autism, Cerebral Palsy, Mental retardation and Multiple Disabilities, Government of India (2005–2015). Sangath has an established history of collaboration with the governments across India for implementation of its research studies, including the Government of New Delhi.

#### Recruitment site

The two recruitment sites are large tertiary centres in New Delhi. The All India Institute of Medical Sciences (AIIMS) is an autonomous institution of national importance. Maulana Azad Medical College and associated Lok Nayak Hospital (MAMC-LNH) is one of the oldest medical centres in India being established in 1930. Both centres are premier medical training institutes for undergraduate and postgraduate education. The Departments of Paediatrics in both hospitals have speciality child developmental clinicians, with a special interest in Autism; in MAMC-LNH, it is the Child Development Centre (CDC) which runs daily services, and at AIIMS, there is an autism clinic once a week within the Child Neurology Division. AIIMS alone had 400 new cases of autism registered in 2015–2016 of which 150 were from the Delhi region; similarly, MAMC-LNH had 90 new cases from the Delhi region in the same period. A 1-year study at AIIMS found a population-appropriate spread of ages at autism identification (15% aged < 3 years, 49% 3–6 years, 7% > 7 years).

### Eligibility criteria {10}

Children aged 2 to 9 years with ASD were recruited from large tertiary level government organisations providing services to children with ASD in the National Capital Territory of Delhi (NCT of Delhi), India.

Inclusion criteria:

1. Children aged over 2 years 0 months and under 10 years 0 months at randomisation. The trial is focused on this age range because scientific and clinical consensus is that early intervention is preferable and the empirical base for our intervention, as with most other interventions for ASD, is for children in the early years [13]. The PASS and PASS + trials have shown feasibility of the intervention across this age range.
2. Clinical diagnosis of ASD and fulfilling ASD criteria on the INCLEN Diagnostic Tool for Autism Spectrum Disorder (INDT-ASD) [14], a ‘Diagnostic and Statistical Manual of Mental Disorder’-based clinical diagnostic algorithm developed in India and validated by our team against specialist assessment
3. Families who reside in the 11 districts of NCT of Delhi

Exclusion criteria:

1. Significant hearing or visual impairment in child or parent
2. Child developmental age of < 13 months equivalent
3. Twins
4. Child with epilepsy with a seizure in the previous 6 months
5. Residence outside the NCT of Delhi
6. Involvement in another research trial
7. Children with other significant genetic, neurodevelopmental or physical disability
8. Current severe learning disability in the parent or current severe parental psychiatric disorder
9. Current safeguarding concerns or other family situation that would affect child / family participation in the trial

These factors make the participation with the intervention unreliable or difficult to deliver.

### Who will take informed consent? {26a}

Informed consent will be taken by COMPASS research associates (RAs) who have received mandatory training on Good Clinical Practice from the University of Manchester. Potential participants will initially be identified and informed by referring clinicians at recruitments sites who will be supported by COMPASS RAs or project appointed recruitments officers. RAs will contact potential participants to take appointments for consenting and simultaneously share a participant information sheet (PIS) digitally. On the appointment day, RAs will contact participants and have a more detailed discussion about the trial, go through the PIS and answer any queries that they may have. Along with this, they will reconfirm the eligibility criteria for inclusion in the trial and proceed to obtain informed consent with eligible families. They will seek permission to audio record the consent and read out the complete consent form. The consent will be recorded on paper, in audio form and on a REDCap application (described below). Following this audio consenting, signatures will be obtained on a paper consent form from the primary caregiver of the child during an in-person visit (either at a centre or at the participant’s home). The consent status for a participant will be marked completed once signatures are obtained in consent forms.

### Additional consent provisions for collection and use of participant data and biological specimens {26b}

No additional consent at present.

## Interventions

### Explanation for the choice of comparators {6b}

The trial will take place in a population representative of the urban disadvantaged, a fast-growing demographic who represent an under-researched and under-served population in India and in global health. Most families in this population access care in the public sector tertiary centres available at minimum cost across the city. The services in two of these centres are our TAU and represent the ‘best available’ care in the public sector. The Department of Paediatrics, AIIMS, runs an autism clinic once a week. Child psychologists at the clinic provide ASD diagnosis and services based on applied behaviour analysis. In addition, parental counselling and training and medical treatment are provided for the management of comorbidities. The Child Development Centre (CDC) at MAMC-LNH is an assessment and treatment centre, which runs 6 days a week. It caters to children with neurodevelopmental disabilities and behavioural and psychological problems. The CDC offers services like speech and language therapy, occupational therapy, physiotherapy, special education, and clinical therapy along with medical management for comorbidities.

Systematic data on precise components of TAU and TAU uptake is currently lacking and will be collected and summarised using data collected to support cost-effectiveness analysis within the trial using a Cost of Illness Inventory (see Sect. 11). We will randomise approximately equal trial participants from the recruitment sites to minimise any bias due to differences in the TAU arms in the two sites.

With regard to the risk of contamination across trial arms, it is unlikely that families in the intervention arm will be close to other families in the TAU arm and unlikely that detailed intervention information would be shared between participants.

### Intervention description {11a}

**Fig. 1:**
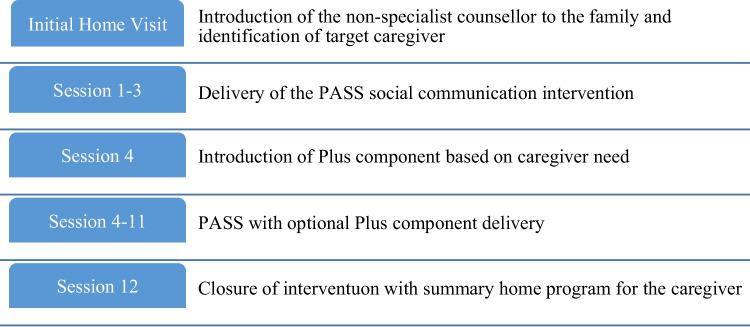
Intervention procedure

The manualised ‘Parent-mediated Autism Social communication Intervention for non-Specialists’ (PASS) is a parent-mediated social communication intervention adapted for the study context from an evidenced UK model. Manualised video-feedback training is used with parents to aid social communication with their autistic child. The video-feedback method is evidenced from other work as a critical active ingredient in this form of therapy [15]. Additional co-morbidity ‘Plus’ (PASS +) modules have been systematically developed as a series of therapeutic modules for the most common coexisting problems, which could be integrated efficiently into the core PASS social communication intervention and which could be delivered with the already proven core social communication intervention (PASS). It includes modules for sensory difficulties, feeding, toileting, sleep and behaviour problems as well as a module to support parent wellbeing (see Fig. 1).

#### Delivery

COMPASS will work with mix of ASHAs and ASHA like workers (collectively referred to as COMPASS Counsellors) who are either already part of the health system or share similar characteristics with the ASHA work. This will ensure that the intervention is scalable through existing human resources. The COMPASS team will work with the State Health Mission to identify ASHA workers spread across a geographical area suitable to our recruitment centres. We will simultaneously recruit ASHA like workers, from the same communities who will be trained and supervised along with the ASHA workers. We will use the same cascade training and supervision model as in the PASS and PASS + trials but scaled up through a comprehensive digital platform.

#### Digital platform

A comprehensive digital platform is being developed through Patel’s Wellcome Trust Principal Research Fellowship-funded PRIDE project: https://www.phfi.org/news-and-events/key-projects/pride. The PASS + intervention is already utilising digital technology to deliver the intervention through video-feedback on tablet computers; the integration of other elements into the PRIDE digital platform will be a major output of the formative phase and a key innovation within this scale-up project. It will allow remote supervision, peer therapy support and targeted training over three separate 3-day periods, with subsequent practice learning and supervision. This platform will thus successfully transition PASS and PASS + into a scalable m-health platform for delivery within existing health systems of an individualised intervention (which is the only kind that has shown success in this complex disorder).

#### Dosage

Families will undertake up to 12 face-to-face communication sessions over 8 months with the COMPASS Counsellors, along with 30 min daily home practice between sessions. PASS and PASS + pilot RCT data have demonstrated the feasibility, acceptability and pilot effectiveness of a 12-session intervention in this environment [9, 10]. Parents will also be offered the Plus modules which address coexisting problems as per the PASS + intervention protocol.

#### Fidelity

All therapy sessions will be audiotaped for the purposes of monitoring fidelity. Variability due to therapist effects will be minimised by frequent clinical supervision. Five percent of randomly selected sessions for each COMPASS counsellor will be formally coded for fidelity against the treatment manual over the course of the study by the UK team using the model successfully used in PASS and PASS + . The unique practice platform we will develop for COMPASS will ensure the highest level of fidelity of the intervention delivery and promises to be the most feasible evidence-based approach for scale-up in LMIC settings.

### Criteria for discontinuing or modifying allocated interventions {11b}

Decision to discontinue trial intervention will be taken by the research team if a serious adverse event (SAE) and/or multiple adverse events (AE), possibly or definitely related to the intervention, were reported by participants, or otherwise for the welfare or safeguarding of participants, where participation is no longer in their best interests. An adverse event will be considered serious when there is participant’s or caregiver’s death, a life-threatening injury requiring hospitalisation, prolongation of hospitalisation, significant disability/incapacity, congenital anomaly or requirement of intervention to prevent permanent impairment or damage. We do not anticipate any SAEs to relate to the trial intervention. Participants can also withdraw from the intervention programme and/or the trial at any time without the need to give any reason. The research team will keep an updated log of all withdrawals with relevant dates and details of reason for withdrawal. Participants will not be replaced and all data collected up until the point of withdrawal will be used unless participants explicitly request removal of their data from the trial.

#### Pandemic-related modifications

Changes have become necessary due to the unprecedented COVID-19 restrictions in India in general and Delhi in particular, during the course of the trial—which made it impossible to deliver the planned face to face sessions, either in the participant’s homes or in any other setting. Moreover, the future pattern of social life in the medium term is unclear in India as it is internationally. From June 2020, an online/remote option for delivery of the PASS Plus intervention has been initiated with practice cases. This is through videoconferencing technology or where this is not possible due to lack of internet availability or access to a smartphone, by telephone. The default mode of intervention delivery will still be face-to-face when possible, but (as with now many health interventions internationally) we need to develop the capacity for a blended model including online remote delivery when needed.

Parents will be taught how to record and upload/send parent–child interaction play videos in advance of the first remote session. Where necessary, an additional introductory session will be included to ensure parents are familiar with and comfortable using the required technology. These procedures have been developed during the recent virtual delivery of this intervention to nine practice families outside the trial. We have found that the process has been acceptable and feasible for parents and therapists for most sessions delivered. They have also given positive feedback on becoming more proficient with using the various digital applications because of the intervention. Regarding supervision, we have found that the transfer of knowledge of strategies appears to be taking place as well as it would have in a face-to-face session.

Making these immediate changes will allow families to continue/commence the intervention, despite the restrictions imposed by COVID-19 lockdowns. Should this online delivery prove successful and acceptable and should the unfolding situation require it, we will then be able to consider future-proofing the trial over the medium and longer term to allow the trial to complete in the face of an uncertain situation. This would then have the further added value of future-proofing the intervention itself for work at scale in India, when it may well be (as internationally) that remote working becomes a standard option for healthcare delivery in the future.

### Strategies to improve adherence to interventions {11c}

See below under ‘Response to COVID-19 pandemic’.

### Relevant concomitant care permitted or prohibited during the trial {11d}

Participants within the intervention and TAU will be permitted to access any concomitant care or intervention.

### Provisions for post-trial care {30}

There are no provisions for post-trial care. COMPASS is a low-risk trial, and no significant harm is anticipated for participants during the course of this trial. There is no provision of compensation to those who suffer any harm from trial participation.

## Outcomes {12}

### Primary outcome

The primary outcome is as follows: autism symptom severity assessed at 9 months post randomisation using the Brief Observation of Social Communication Change (BOSCC) [16], a blinded researcher symptom-coding from video-taped child-researcher interaction. BOSCC is an adaptation of the standard diagnostic symptom algorithm to (i) maximise sensitivity to symptom change in treatment studies, with codes combining symptom frequency and severity on a 12-item, 0–5 scale (total 0–60), and (ii) be delivered and coded by non-specialists, making it highly suitable for LMIC implementation. It shows good reliability, validity and evidence of sensitivity [16]. Feasibility of such video-interaction coding in India was shown in the initial PASS pilot trial [9], and, in the PASS + pilot, BOSCC was used with 100% success at baseline (*n* = 40), and 35 participants successfully completed it at endpoint. In the PASS + pilot, the instrument development team from the US trained master trainers from UK and India (Divan and Cardozo), who undertook an adaptation for the South Asia context (including simplified behavioural descriptions and visual aids). They then trained project-based RAs. Coding of the BOSCC within the trial was shared by two project RAs and a master coder based in the USA. The inter-rater reliability (*n* = 16; comparing 2 trial RAs with the US master coder) gave an ICC of 0.80.

### Secondary outcomes

Parent–child communication will be measured using the Dyadic Communication Measure for Autism (DCMA) [17], a researcher-rated measure of parent–child social communication interaction used in both PASS and PASS + trials. This measures the proximal treatment outcomes reported in PASS, which were also demonstrated to mediate autism symptom outcome in the original UK trial.

Others secondary child and parent measures are listed below.

Child measures:

1. Vineland Adaptive Behaviour Scales, Third Edition (Vineland 3), is a parent-rated measure of adaptive behaviour that is widely used to assess children with intellectual, developmental and other disabilities [18]
2. Communication and Symbolic Behaviour Scales Developmental Profile (CSBS-DP) [19] is a measure of early communication and symbolic skills in young children that aims to identify those who have or are at-risk for developing a communication impairment
3. Development Behaviour Checklist (DBC-P) [20] can be used for the assessment of behavioural and emotional problems of children and adolescents with development and intellectual disabilities. It is a questionnaire completed by parents or other primary caregiver and reports problems over a 6-month period
4. Health status: parent-rated Child Health Utility-9D Index (CHU-9D), valued to allow calculation of QALYs [21]
5. Paediatric Quality of life Inventory [Peds QL (2–4), (5–7), and (8–12)] is a modular approach to measuring health-related quality of life (HRQOL) in healthy children and adolescents and those with acute and chronic health conditions
6. Screening Tools for Autism Risk using Technology (START) developed by Sangath and other collaborators screens for autism risk in community setting using a digital application measuring attention disengagement, social attention and joint attention

Parent measures:

1. Research on Autism and Families in India (RAFIN) [22] developed by Action for Autism, measures knowledge of autism, acceptance of the condition, empowerment and advocacy related to autism amongst parents of children with autism. This was removed in response to the COVID-19 pandemic (see below)
2. Warwick-Edinburgh Mental Well-being Scale (WEMWBS) is a 14-item scale of mental well-being covering subjective well-being and psychological functioning in adolescents and adults, in which all items are worded positively and address feeling and functioning aspects of mental health.
3. EQ-5D is a self-report measure of health status, with a descriptive system comprising five dimensions (mobility, self-care, usual activities, pain/discomfort, and anxiety/depression)

### Participant eligibility

The INCLEN Diagnostics Tool—Autism Spectrum Disorder (INDT-ASD), an indigenously developed tool for the assessment of Indian children, will be used to ascertain symptomology of ASD. Additionally, the Mullen Scale of Early Learning (visual reception and fine motor skills) will be also be used to establish participant eligibility and to characterise the sample’s development levels. This was replaced by the Vineland 3 later in the trial as explained in the following section.

### Baseline measures

Additionally, at baseline data on demographics (including child age, parent age and ethnicity, family SES), clinical information (date of ASD diagnosis, other medical diagnoses, schooling, interventions) and the home environment (including number of people in the household, number and age of siblings, language spoken) will also be collected.

### Service use

The Cost of Illness Inventory (COII) captures costs related to education, childcare, relocation, healthcare contacts (outpatient, inpatient, medical emergencies, investigations and medication), religious retreats and rituals, specialist equipment, workshops and training, special diet, support and care, certification, occupational adjustments and government rebates/schemes [23].

### COVID-19 Impact Questionnaire

The COVID-19 Impact Questionnaire was added after the COVID-19 pandemic to understand the impact of the pandemic and associated lockdowns on the families.

The adverse event form covers reporting of adverse events where there is any safety concern for participants as well as anything relevant to the continuation of the study or where the principal investigators (PIs) feel that the data monitoring and ethics committee (DMEC) should be informed.

### Changes to evaluation procedures due to COVID-19 pandemic

In response to the major disruption throughout India and especially in NCT of Delhi in access to communities and households caused by the COVID-19 pandemic, we implemented changes to research procedures. The intense advocacy for social distancing, perceived health risks and widespread stigma made in-person contacts with trial participants infeasible in the study area. Considering these evolving situations changes in the trial’s assessment procedures were proposed in August 2020 to enable continuation of participant recruitment and assessments.

#### Hybrid evaluation schedule

Majority assessments that were earlier administered in person were planned to be conducted via telephonic interviews. Audio records of informed consent were taken. Detailed procedures were developed for secure storage and sharing of consent information. There were no changes in outcome measures of the trial; however, for ascertaining eligibility in terms of age equivalence, the motor skill domain of Vineland 3 was used as a replacement for Mullen Scales of Early Learning. The RAFIN was dropped given its relative difficulty with administration over phone. A COVID-19 Impact Questionnaire was added to understand the impact of the pandemic and associated lockdowns on the trial families. All assessments were planned over five contacts—four telephonic and one in-person (preferably in the office, if not, then at participant’s home).

Adequate precautions including routine sanitisation of assessment rooms and kits before and after assessments, providing sanitised office transportation to participants, regular temperature checks of all team members and visiting families, supporting COVID-19 tests and vaccination of team members and use of transparent masks during in-person assessments were taken to minimise exposure to COVID-19 for both participants and assessors.

Adapting the evaluation plan allowed trial assessments to continue in a situation where home visits in the community were not possible. Telephonically contacting families reduced the chances of exposure to COVID-19. However, in situations where telephonic interviews were not possible and there was mutual agreement to undertake home visits, assessors visited participants in communities. This has particularly been possible in the second quarter of 2022 which has seen reduction in COVID cases in the NCT of Delhi. Furthermore, continuing evaluations also ensures maintenance of competency of the trained assessors.

#### Adapting assessment timepoints

As per original trial protocol, endline assessments were to be conducted between 9 and 12 months from randomisation. However, due to COVID-19-related lockdowns and restrictions, for a subset of participants who were affected by trial pause, an additional 4 months will be added to this assessment window. Thus, for such participants, the endline assessments will be conducted between 13 and 16 months from randomisation.

For participants whose assessments will not be completed in the given time, an additional month was given; failing which, all such participants were classified as endline missed. These participants were then approached for follow-up assessments.

Follow-up assessments for the trial families were scheduled at 15 months from randomisation. However, similar to the impact of a trial pause on the endline, for a subset of participants, an additional 4 months will be added which shifted the follow-up start point from 15 to 19 months. Additionally, a minimum gap of 3 months will be ensured between the completion of end line assessments and start of follow-up assessments.

#### Trial update as on March 2023

The COMPASS trial completed randomisation of 261 participants on 16 December 2022. As of 31 March 2023, 157 (60%) endpoint assessments were completed and 30 participants (11.5%) were lost to follow-up. Also, 86 (33%) follow-up assessments (15 months post randomisation) were completed by the end of March 2023.

Adaptation to the assessment processes were made during the COVID-19 pandemic-related restrictions (these are detailed the above section). It has taken relatively more time to complete the telephonic assessments compared to in-person contacts. Telephonic assessments take between 4 and 6 contacts with each participating family; every contact takes around 60 min and an additional in-person contact for BOSCC, DCMA and START administration. In-person assessments are completed in 3 contacts, each ranging from 60 to 90 min. In both cases, the number of contacts and the time duration can vary depending on the family’s need and availability. However, it is also important to note that the method of administration of the primary trial outcome, i.e. the BOSCC has remained same throughout the trial (Table 1).

**Table 1:**
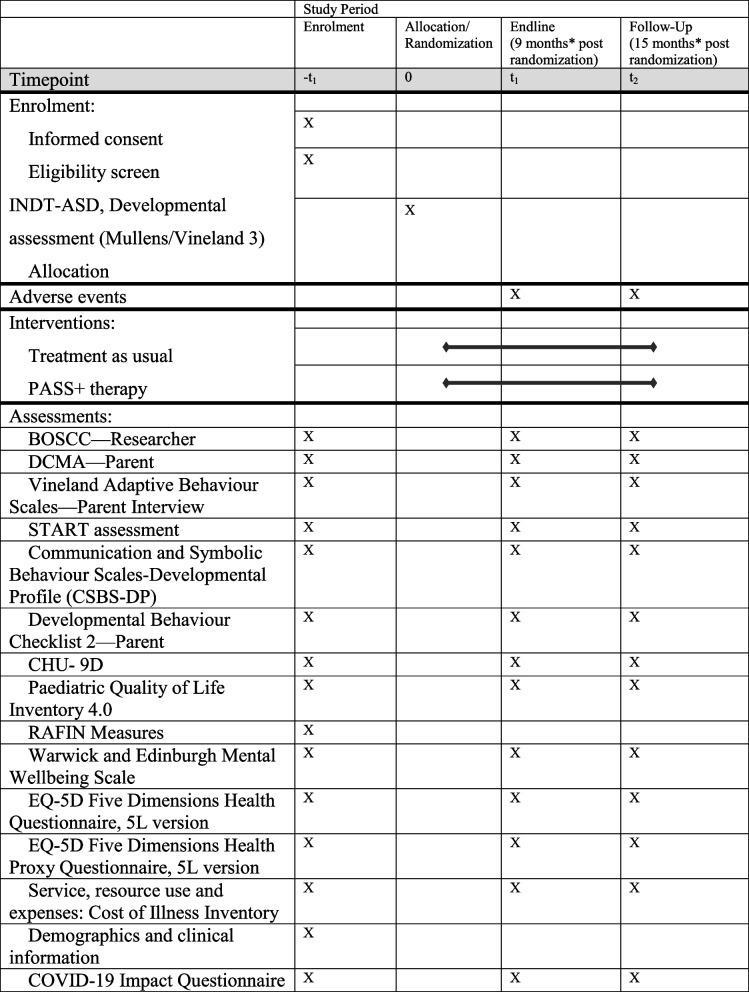
Schedule of assessments (revised to reflect pandemic-related adjustments)

^*^Specific changes made due to COVID-19 related restrictions

## Participant timeline {13}


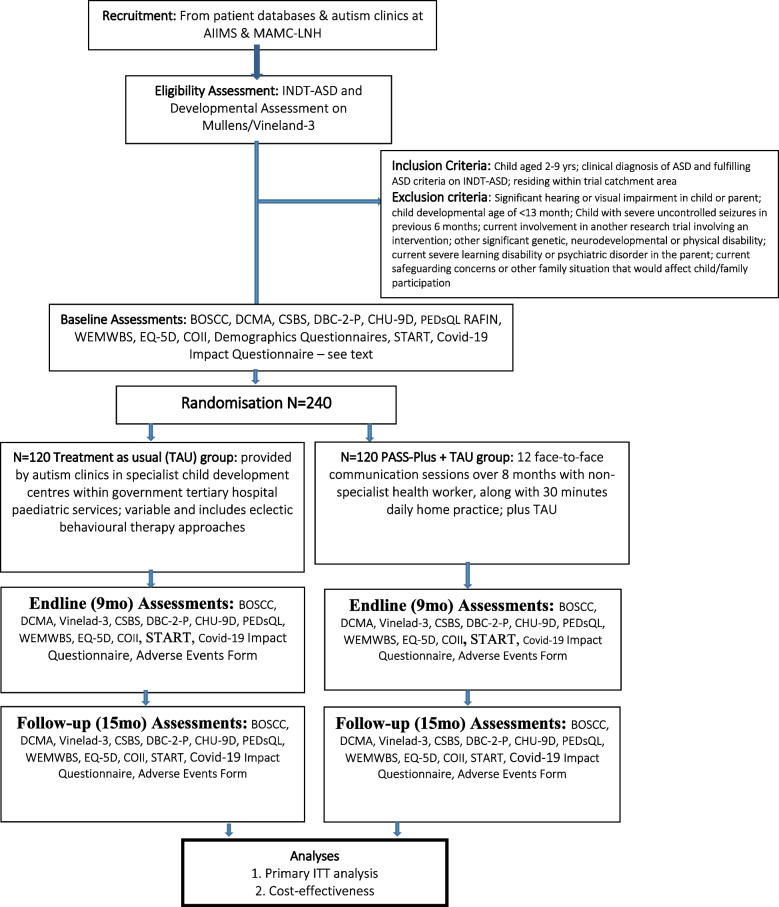


### Sample size {14}

The trial is powered based on data from the PACT, PASS and PASS + trials, along with conservative assumptions on context and implementation. The UK PACT showed an endpoint ES of 0.64 on autism symptoms and 0.55 over 6 years. The PASS trial did not measure autism symptom outcomes, but its effect on the intermediate dyadic interaction outcomes that mediated autism symptom change was equivalent to the PACT study. Given the possible challenges of implementation at scale in India using existing health system workers and health system-like workers, in COMPASS, we hypothesise a conservative target effect size of 0.45 for BOSCC symptoms (representing a clinically relevant 5-point change) [16]. We further assume a conservative intra-cluster correlation of 0.02 to account for variation in therapist quality and clustering amongst the 20 intervention therapists, each seeing on average 8 participants (TAU participants treated as clusters of size 1 with ICC 0) [24]. Given these assumptions, 96 successfully followed up in each group would give 90% power to detect an effect size of 0.45SD and 85% power for effect size of 0.37 SD (two-tailed significance level 0.05 and *t*-test assumed, using Stata ‘clsampsi’). Our pilot studies showed attrition rates leading to endpoint missingness of 3/65 (4.6%) in a semi-urban setting (PASS) and 6/40 (15%) in a rural setting (PASS +). We have modelled a conservative 20% attrition in this study in a large city, giving a recruitment target of 240 patients, making this trial larger than any yet published in the autism psychosocial intervention literature. Mediation effects were shown in the UK PACT trial with a comparatively smaller *n* = 152.

### Recruitment {15}

Both recruitment site hospitals have a database of children with ASD, which will be available to the research team to identify potential participants, with support of a referring clinician at the recruitment site. Enrolment of new cases registering with both sites will also be undertaken. Our planned recruitment rate of 17/month overall will be well within this total of newly registered and historic cases. Families will be initially identified and informed by referring clinicians at recruitments sites who will be supported by COMPASS RAs or project appointed recruitments officers. On agreement this team of clinicians, RAs and recruitment officers will assess geographical eligibility and proceed with informed consent. Each case will be registered and assigned a unique referral ID number.

### Data management {19}

All trial data will be anonymised. A central master file will be held by the trial director at Sangath. This will contain the key linking anonymised participant IDs to personal details.

COMPASS uses multiple platforms across various institutions for trial data management and randomisation. Details of these are as below:

1. REDCap: This is a secure web application for building and managing online surveys and databases. Whilst REDCap can be used to collect virtually any type of data in any environment (including compliance with 21 CFR Part 11, FISMA, HIPAA, and GDPR), it is specifically geared to support online and offline data capture for research studies and operations.
2. REDCap Cloud (RCC): This aggregates real-world data in a standard way and mines it to make unbiased and data-driven discoveries on clinical trials. The RCC helps the clinical trial unit (CTU) at the University of Manchester and Sangath, India (COMPASS Trial Team), to transfer, integrate and monitor data quality.
3. COMPASS Archival Unit (AU): This sub-team of evaluation team members collects, runs quality checks and systematically stores all data collected using paper forms.
4. King’s Clinical Trials Unit (KCTU): The KCTU manages a bespoke online randomisation system, supporting simple randomisation, block randomisation, stratified block randomisation and minimisation. The COMPASS uses KCTU services for randomisation of participants in the trial into the treatment as usual (TAU) group or PASS-Plus + TAU group.

## Assignment of interventions: allocation

### Sequence generation {16a}

Children will be assessed for eligibility using the following: (1) the INCLEN Diagnostic Tool-ASD (INDT-ASD) to confirm the ASD diagnosis and (2) the Mullen Scales of Early Learning [25] to confirm a level of non-verbal development of 13 months or more. As mentioned above, we will use the motor skill domain of Vineland 3 for ascertaining eligibility in terms of age equivalence as a replacement for Mullen Scales of Early Learning as an adaptation to the COVID-19-related restrictions. RAs will then undertake baseline assessment with all consented and eligible participants prior to treatment assignment. Randomisation will be conducted through the King’s Clinical Trials Unit web-based randomisation service. Allocation will be by minimisation, controlling for age strata (2–4 years, 5–9 years) and centre (AIIMS, MAMC-LNH). Randomisation confirmation will be emailed to the trial director in India and to the CTU and treatment allocation will be emailed to the intervention supervisors/coordinators by the trial director.

### Allocation concealment mechanism {16b}

As randomisation will be performed through an independent service at KCTU, the allocation sequence will be concealed until participants are assigned allocations.

### Implementation {16c}

Participants will be recruited and consented into the trial by COMPASS RAs. Eligibility and baseline assessments will be conducted before treatment assignment. Participants will be assigned to one particular randomisation batch. The list of participants for each batch will be sent through to the trial director who will carry out the randomisation. Unblinded treatment allocation emails will be sent to the intervention coordinators. They will then contact each participant via phone call informing them of their allocation. Participants allocated to the intervention group will be invited to the sessions.

## Assignment of interventions: blinding

### Who will be blinded {17a}

Research and statistical staff will be blinded to treatment allocation; therapists, families and parent-rated secondary outcomes cannot be blinded. Primary and researcher rated secondary outcomes will be done by video-coding, by trained coders blind to allocation.

### Procedure for unblinding if needed {17b}

All analysis will be pre-specified and the trial dataset will be generated with a dummy variable for group allocation, and the primary analysis will be conducted prior to unblinding group identities.

## Data collection and management

### Plans for assessment and collection of outcomes {18a}

The primary endpoint will be at 9 months after randomisation, with a follow-up assessment at 15 months with specific COVID-19-related adaptations as mentioned above. Baseline, endpoint and follow-up data will be collected by RAs during home and centre visits. All data will be collected in accordance with trial standard operating procedures. Data for the majority of measures will be captured directly onto a tablet database using REDCap; some data will be collected in paper format if difficult to collect and/or score in REDCap. Data collected using paper forms will be entered directly into the Redcap Cloud (RCC) database.

Following are the four types of processes followed for data management.

#### Type 1

In this, data will be directly collected on REDCap by RAs. The evaluation measures in the type 1 are as follows: PIS, demographics questionnaire, COVID-19 Impact Questionnaire, adverse events form, DBC-P, WEMWBS, PEDS-QL and CHU-9D. Once entered in REDCap, the data will then be uploaded to RCC by a data entry operator (DEO).

#### Type 2

Data for these evaluation measures will be collected on paper forms. The evaluation measures in type 2 are as follows: consent form, INDT-ASD, CSBS-DP, EQ-5D and Vineland-3. The data collected on the consent form and the summary scores of INDT-ASD are entered by the RA on REDCap, whilst data for CSBS-DP and EQ-5D is entered by a DEO. A separate process will be followed for Vineland-3, wherein RAs enter data in Q-global, which further provides domain summary scores. These summary scores will then be entered in REDCap by the DEO. All data will finally be transferred from REDCap to RCC. Post the data entry, paper forms will be submitted back to an archival unit (AU) for systematic and safe storage.

#### Type 3

The only evaluation measure in this type will be the COII. The data will be collected on the paper form by RAs and then submitted to the AU. Upon submission, AU will run the required quality check and then hands over form(s) to the DEO for data entry. The data will be directly entered into RCC by DEO.

#### Type 4

The type 4 includes all video and digital assessments, viz., BOSCC, DCMA, and START. The BOSCC and DCMA videos will be shot and stored in the electronic tablet during centre visit. Post centre visit RAs will submit the videos to AU where a quality check of the videos is done by DEO. Once the videos clear the quality check, they will be uploaded on REDCap and thereon to RCC by DEO, post which assessors will be given the permission to permanently delete the videos from their respective tablets.

Appropriate quality control will be carried out during the trial and before the database lock. Primary analysis of the data will take place by the trial statisticians and chief investigator. Other members of the team will also have access to data and will undertake analysis as appropriate and necessary.

### Plans to promote participant retention and complete follow-up {18b}

This trial will require a significant time commitment from families. Each trial family will need to be visited five to seven times over the course of data collection. Some of these visits will be organised at participant homes to minimise inconvenience to families. In recognition and appreciation of this time commitment, all families will receive a participation token of INR 500 on completion of eligibility assessments whether or not they are eligible for the trial. INR 1000 will be provided on completion of assessments at each assessment timepoint, i.e. baseline, endline and follow-up. This will be a total of INR 3500 for each participant across the course of the trial.

On successful completion of the Vineland-3, an assessment feedback report compiled by the research team, detailing adaptive skills, will be provided to all trial participants at baseline and 15-month follow-up assessment time points. This report will also include the age equivalence of the different sub-domains of adaptive skills, viz, communication, daily living, socialisation and motor skills. Periodic newsletters and customised birthday cards will be also sent out all participants through the duration of the trial to promote engagement of participating families.

Furthermore, conducting assessments at endline and follow-up with participants will need concerted efforts by the evaluation team. Hence, information about participants who are non-contactable over telephone calls will be shared with respective recruitment sites so that they can contact the participants and check their willingness to continue participation in the trial. In the event that the recruitment sites are unable to get in touch with them, a visit to participant’s household by a designated COMPASS evaluation team member/assessor will be planned in order to enquire about participant’s willingness to continue and address any concerns that may have. This will be considered as the last attempt, following which, if the participant refuses, they will be considered as having withdrawn consent or, if still non-responsive, then lost to follow-up for relevant reasons.

### Confidentiality {27}

Data protection and confidentiality procedures will be specified and followed, in keeping with Good Clinical Practice and the General Data Protection Regulation 2018. All video recordings will be made only after written consent has been obtained from parents. Video recordings will be viewed only by members of the COMPASS team and for the purposes of the research and therapy, unless further explicit written consent is obtained. All video and audio recordings will be held securely in a locked cabinet on an encrypted hard-drive and will be shared only via a secure server system hosted in the University of Manchester and in accordance with pre-specified highly secure procedures. All data will be kept confidential, accessed only by the trial team. Personal information may be shared only with parental consent, e.g. with clinicians involved with the family. The only time that personal information will be shared without parental consent is if there are serious concerns about the safety or wellbeing of a child or vulnerable adult. In this event, local procedures for safeguarding children and vulnerable adults will be followed. Electronic and paper-based data collection forms will be identifiable only by participant ID and will contain no names or contact details. Personal and sensitive data will be stored separately and securely in an encrypted form, on a password-protected hard drive or computer in a secure office. If personal information needs to be emailed, this will be in an encrypted form.

## Statistical methods

### Statistical methods for primary and secondary outcomes {20a}

All statistical analysis will be undertaken after the 15-month outcome measures are completed. All analyses will be carried out using the most recent version of Stata [26]. In accordance with CONSORT Statement for Social and Psychological Interventions (2018), we will report all participant flow through the trial. Additionally, we will follow the CONSORT and SPIRIT Extension for RCTs Revised in Extenuating Circumstances (CONSERVE) statement for reporting the results of the trial [27]. Descriptive statistics of recruitment, drop-out and completeness of interventions will be provided. The main efficacy analysis will be via intention-to-treat including all participants, with no planned interim analysis for efficacy or futility. Baseline characteristics will be presented by randomised group without formal statistical tests.

We will test the primary hypothesis for between-group difference in the primary outcome (BOSCC) using generalised linear mixed models allowing for clustering by participants and therapists with fixed effects including baseline outcome measure, centre, age-group stratifier, treatment assignment, time (treated as a categorical variable) and time * treatment interaction. We will explore potential moderating effects by baseline severity, co-morbidities, home environment and parental education including interactions between the moderator and randomisation. Marginal treatment effects will be estimated for outcomes at each time point and reported separately as adjusted mean differences in scores between the randomised groups with 95% confidence intervals and two-sided *p*-values. The secondary outcomes will be analysed using an analogous method.

The analysis will use statistical techniques for handling missing outcome data under a missing at random assumption and simple mean imputation for missing baseline measures.

As a secondary exploratory analysis, we will estimate the effect of each additional adequate therapy session using instrumental variable regression, the model being identified by assuming an exclusion restriction of the form that the offer of treatment does not of itself influence the outcome, once receipt of treatment has been accounted for.

For the mediation analysis, if the efficacy analysis shows significant between group differences in the mediator (DCMA), then we will use parametric regression models to test for mediation of the intervention on BOSCC outcomes through DCMA. Since all the measures are continuous, the indirect effects are calculated by multiplying relevant pathways and bootstrapping is used to produce valid standard errors for the indirect effects. Mediation analyses are potentially biased by measurement error in mediators and hidden confounding between mediators and outcomes; we will build on our previous methodological and applied work in this context to include repeated measurement of mediators and outcomes to account for classical measurement error and baseline confounding.

### Cost-effectiveness analysis

The cost-effectiveness analysis aims to investigate the cost-effectiveness of the intervention compared to TAU. TAU reflects the comparator arm of the trial and is an appropriate comparator as it reflects standard practice in the local setting [28]. The cost-effectiveness analysis will use a societal perspective, with a 15-month time horizon. Costs will be estimated from COII at baseline and 9- and 15-month follow-up. The costs of providing the intervention will be derived from a detailed process evaluation, to estimate the costs of training, delivery and supervision and take account of barriers experienced during the trial such as attrition of the front-line workers and variable periods taken to attain competency. Costs of scale up of this intervention (if found effective) will also be estimated by use of appropriate level of remuneration for staff involved in the intervention. The analysis will use the adapted CHU- 9D Index and associated utility weights to estimate quality-adjusted life years (QALYs) for the child. Costs and outcomes will not be discounted due to the trial time horizon and timing of follow-up assessments.

Regression analysis, adjusted for key covariates, will estimate the net costs and QALYs of the intervention. The estimates of net costs and QALYs from the regression analyses will be bootstrapped to simulate 10,000 pairs of incremental cost and QALY outcomes of the COMPASS intervention [29]. This will include the following: (i) plotting the distribution of pairs of net costs and QALYs on a cost-effectiveness plane, to assess parameter uncertainty, (ii) generate a cost-effectiveness acceptability curve to estimate whether the additional cost of a QALY gained by the COMPASS intervention is acceptable to decision makers; (iii) estimate the probability that the COMPASS intervention is cost-effective compared to TAU (iv) estimate a net benefit statistic. Sensitivity analyses will explore the intervention’s cost-effectiveness using alternative measures of child health benefit, parental health benefit, the perspective of funders of health, education and social support services. Whilst 1 to 3 × GDP per capita has commonly been used as the cost-effectiveness threshold within studies in India, this has been noted to be high. More recent evidence estimated the cost-effectiveness threshold on the basis of health expenditures per capita and life expectancy at birth (resulting in estimates of $487 ($249–$618) [30, 31]. Due to uncertainties around the cost-effectiveness threshold in India, a range of cost-effectiveness thresholds will be presented using the latest evidence at the time of analysis.

The economic evaluation will be reported in line with the Consolidated Health Economic Evaluation Reporting Standards (CHEERS) statement [32].

### Interim analyses {21b}

There was no planned interim analysis for efficacy or futility.

### Methods for additional analyses (e.g. subgroup analyses) {20b}

There are no planned sub-group analyses at this time. If any become necessary, they will be pre-specified in the statistical analysis plan.

### Methods in analysis to handle protocol non-adherence and any statistical methods to handle missing data {20c}

The economic analysis will use a within-trial, intent to treat approach. Missing data will be accounted for in the analyses of net costs, net QALYs and cost-effectiveness acceptability. The methods used to deal with missing follow-up data will be determined according to the extent and pattern of missing data (e.g. multiple imputation, missing indicator or propensity score methods) [33–35].

### Plans to give access to the full protocol, participant-level data, and statistical code {31c}

There will be a data sharing and monitoring plan to manage access to trial data post-trial from external researchers. The data custodian will be professor Jonathan Green, chief investigator of the study.

### Plans for collection, laboratory evaluation, and storage of biological specimens for genetic or molecular analysis in the current trial and for future use in ancillary studies, if applicable {33}

COMPASS will not collect any biological specimens.

## Oversight and monitoring

### Composition of the coordinating centre and trial steering committee {5d}

A trial steering committee (TSC) was assembled which includes an independent chair (Dr. Shoba Srinath, child psychiatrist, ex prof and head of child psychiatry, National Institute of Mental Health and Neurosciences, Bengaluru, India), a parent representative (Madhusudan Sriivas), a representative of the National Health Systems Resource Center (Dr. Rajani Ved [2018–2022] and Dr. M A Balasubramanya [2022 onwards]) and an experienced community-based paediatrician (Sunanda Kolli-Reddy). In 2019, Mr. Shamika Ravi, a health economist working in New Delhi, was invited to join the TSC. In 2020, the TSC was expanded to include the director, Department of Health and Family Welfare and State Nodal Officer (Delhi State Health Mission), and paediatrician Dr. Monica Rana, whilst in 2022, the international triallist Prof. Alan Stein, child and adolescent psychiatrist from the University of Oxford, was invited to the committee. The TSC has been consulted on the design, protocol, techniques for ascertainment and measurement. The TSC meets at least once prior to the commencement of the trial and at least annually thereafter.

### Composition of the data monitoring and ethics committee, its role and reporting structure {21a}

The data monitoring and ethics committee (DMEC) has been formed which includes Dr. Mayada Elsabbagh (DMEC Chair), Dr. K John Vijay Sagar (DMEC member) and Prof. Bhaswati Ganguli (DMEC member). The DMEC meets at least once annually during the course of the trial.

### Adverse event reporting and harms {22}

We will collect information about adverse events at each follow-up visit and record adverse events in a standard format. Adverse events will be monitored by the DMEC and TSC. Serious adverse events (SAEs) will be reported to the project management group and sponsor. If any of the SAEs are a suspected unexpected reaction to the therapy (it is acknowledged that this is highly unlikely in this trial), these will be reported immediately to the sponsor, ethics committee and DMEC.

### Frequency and plans for auditing trial conduct {23}

Trial conduct and compliance with the protocol and standard operating procedures (SOP) will be monitored by with annual audits from the sponsor and annual reports to ethics committees and funder.

### Informed consent materials {32}

Written, audio-recorded informed consent will be obtained from all families with children with ASD to participate in all trial procedures (uploaded as supplementary material).

### Dissemination plans {31a}

The results from this trial will be submitted for publication in high impact peer-reviewed journals targeted at general and specialist readership. Summary results will be shared with participants through newsletters at the end of the trial. Information about key learnings from the trial will be also shared with caregivers of children with ASD through collaborative sessions organised at the two trial recruitment centres, other non-governmental organisation, parent advocates and community health workers. Dissemination will be done through workshops and conferences at the national and international level to share key learnings and strategies to take the intervention to scale. Also, modelling of costs and benefits of scaling up will be shared if the trial is found to be effective.

## Discussion

This trial builds upon existing research evidence generated both in the UK and South Asia and addresses a key gap within evidence-based and scalable care pathways for autistic children and their families. It is the first trial conducted internationally which aims to provide a robust and well-powered evaluation of an intervention programme for autistic children and their families delivered by non-specialist health workers. If shown to be clinically and cost-effective, this programme will fill an important gap within current care pathways within low-resource contexts. The trial includes blind-rated distal and proximal outcomes assessing communicative and social development, as well as secondary outcomes relating to child functioning and caregiver and child wellbeing. Health economic analysis will provide important information about the costs and benefits of the PASS-Plus programme over and above standard care, as well as presenting a picture of standard care within New Delhi. The trial has run through the COVID-19 pandemic of 2020–2022 when significant social distancing restrictions were in place within India. Modifications were applied in response, which have allowed the trial to continue during much of this period; these have included adjustments to timelines and remote delivery of the intervention and research assessments, as necessary.

## Trial status

Protocol version number 4 dated 03 August 2022.

Date recruitment began: 23 December 2019.

Date recruitment completed: 16 December 2022.

## Data Availability

Primary analysis of COMPASS data will be conducted by the trial statisticians. Health economic data (resource use and utility measures) will be analysed by the trial health economists. Other members of COMPASS team will also have access to data in order to perform any required analysis as per the publication policy.

## Abbreviations

AIIMS: All India Institute of Medical Sciences
AMD: Adjusted mean difference
ANM: Auxiliary nurse midwifes
ASD: Autism spectrum disorder
ASHA: Accredited social health activist
AU: Archival Unit
BOSCC: Brief Observation of Social Communication Change
CDC: Child development centre
CFR: Code of Federal Regulations
CHEERS: Consolidated Health Economic Evaluation Reporting Standards
CHU-9D: Child Health Utility-9D Index
CI: Confidence interval
COII: Cost of Illness Inventory
COMPASS: Communication-centred Parent-mediated treatment for Autism Spectrum Disorder in South Asia
CONSORT: Consolidated Standards of Reporting Trials
COVID-19: Coronavirus disease of 2019
CSBS-DP: Communication and Symbolic Behaviour Scales Developmental Profile
CTU: Clinical trial unit
DBC-P: Development Behaviour Checklist for Parent
DEO: Data entry operator
DHSC: Department of Health and Social Care
DMEC: Data monitoring and ethics committee
EQ-5D: EuroQol-5 dimension
ES: Effect size
FCDO: Foreign, Commonwealth & Development Office
FISMA: Federal Information Security Management Act
GDPR: General Data Protection Regulation
HIPAA: Health Insurance Portability and Accountability Act
HRQOL: Health-related quality of life
IAPT: Improving Access to Psychological Therapy
ICMR: Indian Council of Medical Research
IIAPT: Improving Access to Psychological Therapy
INDT-ASD: INCLEN Diagnostic Tool for Autism Spectrum Disorder
INR: Indian Rupees
ITT: Intention-to-treat
KCTU: King’s Clinical Trial Unit
LMIC: Low- and middle-income country
MAMC-LNH: Maulana Azad Medical College and associated Lok Nayak Hospital
MRC: Medical Research Council
NCT: National Capital Territory
NGO: Non-government organisation
NICE: National Institute for Health and Care Excellence
NIHR: National Institute for Health Research
PACT: The Pre-school Autism Communication Therapy
PASS: Previously called the ‘Parent-mediated intervention for Autism Spectrum Disorders in South Asia’ and now renamed the ‘Parent-mediated Autism Social Communication Intervention for non-Specialists’
Peds QL: Paediatric Quality of Life
PI: Principal investigator
PIS: Participant information sheet
PRIDE: PRemIum for aDolEscents
PUHC: Primary urban health centres
QALYs: Quality-adjusted life years
RA: Research associates
RAFIN: Research on Autism and Families in India
RCC: REDCap Cloud
RCT: Randomised control trial
REDCap: Research Electronic Data Capture
SAE: Serious adverse events
SD: Standard deviation
SOP: Standard operating procedures
START: Screening Tools for Autism Risk using Technology
TAU: Treatment as usual
TSC: Trial steering committee
WEMWBS: Warwick-Edinburgh Mental Well-being Scale
WHO: World Health Organisation
WT: Wellcome Trust
